# Former Foodstuff Products in *Tenebrio Molitor* Rearing: Effects on Growth, Chemical Composition, Microbiological Load, and Antioxidant Status

**DOI:** 10.3390/ani9080484

**Published:** 2019-07-25

**Authors:** Simone Mancini, Filippo Fratini, Barbara Turchi, Simona Mattioli, Alessandro Dal Bosco, Tiziano Tuccinardi, Sanjin Nozic, Gisella Paci

**Affiliations:** 1 Department of Veterinary Sciences, University of Pisa, Viale delle Piagge 2, 56124 Pisa, Italy; 2 Interdepartmental Research Center “Nutraceuticals and Food for Health”, University of Pisa, Via del Borghetto 80, 56124 Pisa, Italy; 3 Department of Agricultural, Food and Environmental Science, University of Perugia, Borgo XX Giugno 74, 06100 Perugia, Italy; 4 Department of Pharmacy, University of Pisa, Via Bonanno 6, 56124 Pisa, Italy

**Keywords:** mealworm, edible insects, animal protein, by-product, entomophagy

## Abstract

**Simple Summary:**

Insects represent a possible alternative nutrient source for food and feed purposes. Insects could be reared on a feed basis alternative to conventional ones of animal origin and could help to face the rising demand of proteins. Mealworm could be reared directly on former foodstuff products allowing to reduce waste materials and enhance profits in several sectors. This study demonstrates that *Tenebrio molitor* rearing can be done on leftovers and by-products with proficient outcomes and high-quality final products. However, rearing substrates must be carefully selected in order to maximize the outcomes in relation to the prefixed goals.

**Abstract:**

*Tenebrio molitor* (mealworm) larvae represent one of the most interesting edible insects and could be reared on alternative feeds, such as former foodstuff products (FFPs). In the present work, five different FFPs (brewery spent grains, bread and cookie leftovers, and mixes of brewer’s spent grain or bread with cookies) were employed as feeding substrates. Larvae’s growth performances, chemical composition, microbial loads, and antioxidant status were determined. Chemical compositions of the substrates affected all the tested parameters. Brewery spent grains-fed larvae showed a faster growth period and higher crude protein and carbohydrate contents. The use of cookies as a single substrate or their addition to spent grains or bread increased the lipids contents, while growth was delayed. Microbial loads were partially affected by the fed diet. The antioxidant status of larvae showed different concentrations of tocopherols isoforms (δ, γ, α) in relation to the diet; however, no differences were detected in relation to the global antioxidant capacity (2,2-azinobis-(3 ethylbenzothiazoline-6-sulfonic acid), ABTS reducing activity; 1,1-diphenyl-2-pircydrazyl, DPPH radical scavenging activity; ferric reducing ability, FRAP). Results point out a high plasticity of mealworm larvae and the potential to tailor the final outcomes in relation to the substrate employed. Mealworms could be practically reared on FFPs to produce food-feed with high nutrient values.

## 1. Introduction

In the light of the increasing world population and the demand of proteins from the developing countries, insects could represent one of the more suitable answers [1]. Insects, compared to conventional production animals, have a lower environmental impact (mostly linked to greenhouse gas production, use of water, and use of arable land); moreover, due to their higher reproductive capacity, nutritional quality, and feed conversion efficiency, they could be taken into account for both food and feed purposes.

Despite researchers’ interests in edible insects, western consumers seem to be cautious to practice entomophagy, mainly in relation to disgust related to western socio-cultural ideas of these animals [2,3]. During the last years, several research studies were carried out to determine which items could be positively accepted by consumers in order to introduce edible insects on the market and increase their use both as food and feed. The main drivers which induced an increase of familiarity and a decrease in the awareness in relation to entomophagy have been identified with the visual presence of insects (visible/invisible, powder/whole), the familiarity with the products containing insects, the correct information about nutritional values, and possible positive effects of edible insects from sustainability and environmental perspectives [4,5,6]. With respect to these last perspectives, the rearing conditions could play a major role, as insects can be reared on sustainable feeds, such as waste or by-products that do not meet the nutritional values needed for the rearing of other farmed animals [7]. Former foodstuff products (FFPs) are defined by the European Commission in the Commission Regulation (EU) No 68/2013 of the catalogue of feed materials as “foodstuffs other than catering reflux, which are manufactured in full compliance with EU food law but are no longer intended for human consumption for practical and logistical reasons or due to problems in manufacturing or packaging which are unlikely to cause any health risks when used as feed”. Therefore, in this category, both leftovers from the food industry mainly composed of bakery products, such as bread, and leftovers composed of confectionery products, such as cookies, are listed. The use of FFPs as feed leads to a decrease in waste material and pollution and represents an interesting opportunity in terms of the circular economy [8]. Thus, FFPs represent a valid and cheap source of energy, and they could be used in insect feeding concerning their essential nutrients [9].

Based on the above-mentioned considerations, the use of FFPs in the rearing of edible insects seems a good compromise to reduce food waste materials, produce valuable new food/feed products, and have a positive impact on the consumers’ perception. However, it has to be mentioned that, even in the insect production sector, there are some limitations for the employment of certain feeds as the diet composition could affect the insect’s development rate and body nutrient composition [7,10].

The mealworm (*Tenebrio molitor* L. 1758; Coleoptera Tenebrionidae) represents one of the most studied edible insects, both as food and feed. The mealworm is a holometabolic insect (complete metamorphosis) and probably originated in the Mediterranean region, but it is nowadays cosmopolitan in its distribution. Due to its low rearing request, the mealworm is one of the promising candidates for an industrial scale production. Indeed, in the last years, several farms and food-feed companies worldwide have started to employ this insect. Nutritional composition of mealworm larvae revealed a relatively high content of protein, 50% on a dry-matter basis [11,12], and lipids, about 30%–34% on a dry-matter basis [12,13,14], with a good composition in amino acids (good source of the essential amino acids), vitamins (i.e., vitamin E, vitamin B12, niacin, riboflavin, pantothenic acid, and biotin), and minerals (P, K, Mg, Zn, and Mn) [15].

Few research studies were carried out on the rearing conditions and on the effects of different substrates on the development and chemical-biological characteristics of mealworm larvae [7,10,13,14,16,17]. Most of the cited studies highlighted a plasticity of *T. molitor* in relation to the substrate, with variations of both development times and nutritional values of the larvae. However, researchers employed mixes of several ingredients as substrates in their studies, since the main focus was to determine the effects on combinations of high/low amounts of proteins, lipids, or carbohydrates. In this scenario, it is very difficult to assess if mealworms could be reared on FFPs directly gathered from the producers (one single type of FFP or a mix of a few). Indeed, as insect rearing does not request specific features in geographical or natural environmental conditions (as carried out in indoor controlled conditions), it is possible to speculate that new farms could be located near the substrate suppliers, and feed manipulation will be kept at a minimum in order to maintain low costs.

Hence, the main aim of this research study was to evaluate the use of three FFPs in *T. molitor* rearing in order to produce larvae for food/feed purposes. In particular, larval development, chemical characteristics, and the microbiological and antioxidant status were determined in mealworm larvae fed with brewery spent grains, bread and cookie leftovers, and on mixes of brewer’s spent grain or bread with cookies (mixed to increase brewer’s spent grain or bread diets in lipid content).

## 2. Materials and Methods 

### 2.1. Diet Preparation

The mealworms were fed with five different diets: brewery spent grains (SG), bread (B), cookies (C), 50% brewer’s spent grain and 50% cookies (SG-C), and 50% bread and 50% cookies (B-C). Spent grains were directly collected from a local brewery and immediately frozen at −20 °C. Bread and cookies were collected from a market shop as daily remains (bread) or soon to expire products (cookies). Excessive humidity of spent grains, bread, and cookies was removed in an oven at 90 °C until the product was completely dry. Spent grains were previously thawed for 18 h at 4 °C. The proximate composition of SG, B, and C is reported in Table 1. The three substrates were then finely ground, and the five diets were formulated.

### 2.2. Insect Rearing and Growth Performances

The mealworms were reared in plastic containers (39 × 28 × 14 cm) at the Department of Veterinary Sciences (University of Pisa, Pisa, Italy) under a laboratory scale production. The temperature was maintained at 25 ± 1 °C with 50%–60% of relative humidity. In the first stage of the trial, 15 boxes were employed (three per diet), in which adult beetles (one to two weeks old) were placed for one week to deposit eggs. Then beetles were removed, and the larvae were left to grow in the substrate. Experimental diets were added weekly if needed (*ad libitum*, weighed before adding), and in order to provide moisture, potatoes slices were deposited once a week. Boxes were visually evaluated three times per week to check larvae health and to remove dead ones.

The mealworms were harvested when the first pupa was observed or after one year of rearing time (in order to maintain a profitable low cost). The development time was calculated between the first day of the experiment and the day of harvesting. Every week, representative samples of larvae (100 per box) were weighed to quantify the growth performance. After harvesting, the larvae were starved for 24 h (apart from samples for microbiological analyses performed with and without starving, see below) in sterile plastic containers with plastic web on the base in order to separate feces and to avoid fecal contact.

The feed conversion ratio (FCR) and efficiency of conversion of ingested food (ECI) were calculated assuming that all the provided feed was consumed as reported by Oonincx et al. [7]. Furthermore, the nitrogen conversion efficiency (N-ECI) was also calculated as reported by Oonincx et al. [7].

### 2.3. Proximate Composition of Feed and Larvae

Proximate composition analyses were performed in triplicate for each sample. Larvae were ground in a blender before being analyzed.

The dry matter content was determined by dehydration in a drying oven at 105 °C until constant weight. The lipids content was quantified by the Soxhlet method using petroleum ether as a solvent. The ash content was determined by incineration in a muffle furnace at 550 °C. The crude protein content was determined by the Kjeldahl method, two protein-to-nitrogen conversion factors were used: 6.25 as normally calculated for meat samples and 4.76 as suggested by Janssen et al. [20] for insects. Carbohydrates were calculated as: 100 − crude protein − lipid content – moisture − ash.

### 2.4. Microbiological Analyses

For microbiological analyses both un-starved and starved larvae were employed in order to quantify the effect of fasting. Substrates were also analyzed, and microbiological amounts lower than the detection limits were reported.

Ten grams of larvae were weighed in sterile stomacher bags, and then 90 mL of sterile saline solution was added. The mixture was homogenized for 60 s in a stomacher (Stomacher^®^ 400 Circulator, VWR International Sr, Milan, Italy). Ten-fold serial dilution series were performed and plated on different media.

Plate count agar (PCA) was employed for the quantification of the total viable aerobic count (incubated at 30 °C for 72 h) and aerobic bacterial endospores after heating the 10:1 dilution at 80 °C for 10 min and performing ten-fold serial dilutions (incubated at 30 °C for 72 h); violet red bile glucose agar (VRBGA) was used to enumerate Enterobacteriaceae (incubation at 37 °C for 24 h); Tryptone Bile X-Glucuronide medium (TBX) was employed to quantify *Escherichia coli* (42 °C for 24 h); Baird Parker medium (BP) was employed for the enumeration of *staphylococci* both presumptive coagulase-positive and -negative (incubated at 37 °C for 24–48 h); yeast extract, dextrose, chloramphenicol medium (YEDC) was used for yeast and mold counts (incubation at 25 °C for 120 h); de Man–Rogosa–Sharpe agar (MRS) was employed to count lactic acid bacteria (37 °C for 48 h in anaerobic conditions); Bacillus Cereus MYP agar was used for the evaluation of the presence of presumptive *Bacillus cereus* (37 °C for 24 h). Absence of *Listeria monocytogenes* and *Salmonella* spp. in 25 g was assessed according to ISO 11290 and ISO 6579, respectively.

All culture media and supplements were purchased from ThermoFisher Scientific, (Milan, Italy) except for Bacillus Cereus MYP agar, which was purchased from Biolife (Milan, Italy). 

Microbial counts were expressed in log colony-forming unit (CFU)/g as mean of three replicates (when one or more samples showed values below the detection threshold, 1.0 log CFU/g, the detection threshold divided by two, 0.5 log CFU/g, was used to calculate the mean and standard deviation as reported by Stoops et al. [21]).

### 2.5. Antioxidant Status

Antioxidant activity of the larvae was quantified as reported by Mancini et al. [22] for meat samples with minor modifications. Three grams of larvae were homogenized in 10 mL ethanol, followed by a centrifugation and filtration (Whatman number 4 filter paper) steps. The filtrate was used to measure 2,2-azinobis-(3 ethylbenzothiazoline-6-sulfonic acid) (ABTS) reducing activity, 1,1-diphenyl-2-pircydrazyl (DPPH) radical scavenging activity, and ferric reducing ability (FRAP). The results of antioxidant capacity were expressed as mmol of Trolox equivalent per kilogram of sample (calibration curves obtained with Trolox at 0–2000 μM, final concentrations).

The tocopherol (δ-, γ-, α-isoforms) contents of the samples were quantified by a high performance liquid chromatography (HPLC) system, according to Zaspel and Csallany [23]. About 3 g of larvae were saponified in 60 g/100 mL KOH in ethanol in a thermostat bath at 70 °C for 30 min. Then the content was sonicated and extracted twice with 15 mL of n-hexane/ethyl acetate (9:1, v/v). After collecting the upper phase, the samples were nitrogen dried and then reconstituted in 200 µL of acetonitrile. Fifty 50 µL was injected into the HPLC system (Perkin Elmer series 200, equipped with an autosampler system, model AS 950–10, Tokyo, Japan) on a Synergy Hydro-RP column (4 µm, 4.6 × 100 mm; Phenomenex, Bologna, Italy). An Fluorometric detector (FD) (model, FP-1525; Jasco, Tokyo, Japan; excitation and emission wavelengths of 295 and 328 nm, respectively) were used to identify the different isoforms. External calibration curves were used to quantify isoforms by increasing amounts of pure tocopherols in ethanol.

### 2.6. Statistical Analysis

The data obtained from proximate composition, microbial determinations, antioxidant capacity, and antioxidant compounds were statistically analyzed using a one-way ANOVA with regards to the different diets (SG, B, C, SG-C, and B-C). The effect of starvation (starved and un-starved) was tested against each different microbiological quantification by a Student’s *t*-test. Statistical significance was set at 0.05 and differences were assessed using Tukey’s test. Linear regression and second-order polynomial quadratic equation were performed to evaluate the effect of each diet on the growth performance. Principal components analysis (PCA) was performed on proximate composition (protein conversion factor of 4.76), microbial loads (fasted samples), growth performance (g weight gain per day), antioxidant capacity (ABTS, DPPH, and FRAP), and antioxidant compounds (tocopherol δ-, γ-, α-isoforms); all the data were mean centered and scaled to a unit standard deviation before analysis. R free statistical software was used [24].

## 3. Results and Discussion

### 3.1. Growth Performances and Proximate Compositions

Proximate compositions of the larvae reared on the different substrates are reported in Table 2.

Dry matter, ether extract, and crude protein contents were affected by the diet. Dry matter of the SG-C larvae was the highest, followed by that of the C and B-C diets. From these data, it would seem that the presence of the cookies induced a decrease of humidity in the larval body, as the larvae fed with spent grains and bread alone (SG and B, respectively) showed the lowest values of dry matter. Cookies also played a major role in the lipid content of the larvae. Diets C and B-C showed the highest contents, followed by B. Spent grains contained low amounts of lipids (3.29%), and consequently larvae fed with SG and SG-C showed lower values compared to those fed other diets.

The highest crude protein contents were shown by diets which included spent grains (SG and SG-C) with minor differences. This value was lower in diets with bread (B and B-C). In this case, the low amount of crude proteins of the cookies (6.55%) negatively affected the chemical composition of the larvae with a consequent lowest body protein content.

As the dry matter analyses showed to be significantly affected by the diet, the proximate composition was also reported as % of dry matter (Table 2). Expressing the data as % of dry matter, the ether extract content showed that B, C, and B-C had the highest lipid contents, followed by SG-C and SG, respectively. Thus, the effect of the cookie fat content was confirmed but the bread diet (B) also played a key role.

Crude protein contents, as % of dry matter, showed only minor differences in the statistical analyses if compared to the expression as % of fresh sample. The conversion factors of 6.25 or 4.76, as well as the expression as % of fresh sample or % of dry matter, drastically affected the final numerical expression; nevertheless, as no standardization is currently used in the insect research field, we choose to report all the data expressions.

Carbohydrates were higher in larvae fed SG and SG-C diets than those fed the other diets. Interestingly, SG had the lowest amount of carbohydrates within the employed substrates. Particularly, moisture and lipids content seemed to affect the calculations of carbohydrates in larvae reared in SG and SG-C.

Chemical compositions of mealworm confirm the data already published [7,10,20] and in particular that body composition showed a considerable plasticity in relation to the diet [7,10]. Notably, in our trial, mealworms fed cookies (C) showed a high amount of lipids (50% on DM base) mostly related to the detriment of the protein content. This data could be interesting as edible insects are almost exclusively recommended as a protein source even though they could contain a high amount of fats.

**Figure 1:**
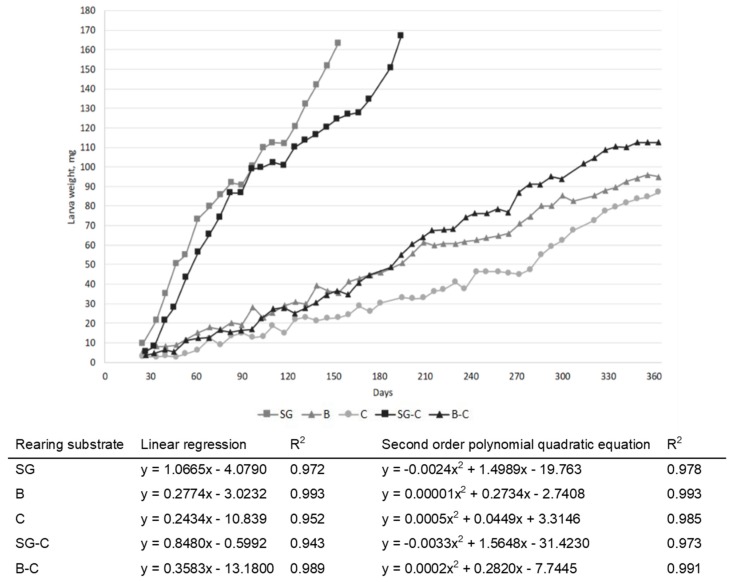
*Tenebrio molitor* larval growth in relation to the diet (SG: brewery spent grains; B: bread; C: cookies; SG-C: 50% spent grains, 50% cookies; B-C: 50% bread, 50% cookies).

The larvae weights are reported in Figure 1; larvae fed SG and SG-C reached the harvesting day approximately after 5 to 6.5 months. After one year of rearing, larvae fed diets with lower content of protein, C, B, and B-C did not reach the pupae stage; therefore, in order to not exceed in the rearing time, their growth was stopped. Larvae fed diets C, B, and B-C reached the final weights of 87, 95, and 112.5 mg, respectively. In linear regression and second-order polynomial quadratic equations (Figure 1), the coefficient of determination indicates that the models explain a high percentage of the variability (R2 between 0.94 and 0.99). Between linear regression and second-order polynomial quadratic equation, the second seems to better fit the larval growth rate.

The FCR (calculated on fresh basis), the ECI, and the N-ECI were severely affected by the dietary treatments. Dietary efficiency of mealworm larvae was strictly related to the chemical composition of the substrate; indeed, larvae fed SG and SG-C showed the lowest values of FCR, 2.22 and 2.76 respectively, confirming the extremely positive potential of this species to convert feed into body weight. On the other hand, larvae fed B, C, and the mix B-C showed FCR values about 8.86, 7.31, and 4.02, which are similar to those of conventional production animals (4.0 for pork and 8.8 for cereal beef; [9]). The variability in FCR values was related to the protein contents of the diets; indeed, SG and SG-C showed the highest amounts of protein. A large variability of the FCR value in relation to the protein content was also reported by Oonincx et al. [7] and van Broekhoven et al. [10] in mealworms fed different diets (from 4.1 to 19.1 and from 2.62 to 6.05, respectively).

The ECI values were higher for SG and SG-C diets (15.85 and 13.94, respectively) than for B, C, and B-C ones (3.79, 4.87, and 8.92, respectively). These data are comparable with those reported by Oonincx et al. [7] and van Broekhoven et al. [10]. As reported before for FCR, the ECI values were affected by the protein content of the diets, which confirms that mealworm efficiency could be modelled through the rearing substrate’s chemical composition.

Nitrogen was more efficiently converted than the other diet components in almost all diets, as in the SG, SG-C, C, and B-C diets, the N-ECIs were higher than the ECI (59.11, 71.75, 13.83, and 14.05, respectively). The diet exclusively composed of bread (B) showed the lowest value of N-ECI (2.77), which is lower than the ECI value. This means that mealworm fed only bread did not convert N of the substrate efficiently into body mass [7]. Indeed, larvae fed only cookies (C) reached approximately the same amount of crude protein content (Table 2) even though the substrate showed to contain half the protein (6.55% in C vs. 11.15% in B, Table 1). The high N-ECI showed by SG and SG-C larvae represents a starting point to reach a relevant benefit of insects over conventional production animals [25].

### 3.2. Microbiological Analyses

Microbial determinations of starved and un-starved larvae are reported in Table 3. 

In the un-starved larvae, staphylococci, yeast-molds, and bacterial endospores were significantly different in relation to the diet. Staphylococci and yeast-molds loads were higher in larvae fed B, C, and B-C, followed by SG-C and SG. Minor differences in the staphylococci amount was highlighted between SG and SG-C in relation to the presence of the cookies. Bacterial endospores were absent in SG, and their presence was related to B and C diets. Indeed, feeding the larvae with a mix of SG and C induced an increase of these bacteria. The highest number of bacterial endospores was detected in the B-C diet, followed by B and C substrates.

Microbial analyses highlighted the total absence of *Escherichia coli* and *Bacillus cereus*, as well as the absence in 25 g of *Listeria monocytogenes* and *Salmonella* spp. Other research studies reported the absence of these bacteria in edible insects reared for human consumption [26,27,28,29].

In starved larvae, diets affected only yeast-molds and bacterial endospores, showing a similar trend of that reported for the un-starved larvae with minor differences. Starvation was not effective in larvae fed SG and SG-C diets, while in larvae fed B, C, and B-C it partially affected the microbial flora. Total viable aerobic counts and lactic acid bacteria were not affected by the starvation. Staphylococci and yeast-molds amounts were significantly decreased in B, C, and B-C fed larvae. Starvation in larvae fed C induced a significant decrease in the Enterobacteriaceae amount, as well as in bacterial endospores of larvae fed C and B-C.

Studying larvae fed wheat bran supplemented with carrots, Wynants et al. [30] reported that fasting for 24 or 48 h, both with and without fecal contact of the larvae, did not significantly affect total viable aerobic bacteria, Enterobacteriaceae, aerobic bacterial endospores, psychrotrophic aerobic bacteria, and yeast and mold amounts.

Contrarily, Mancini et al. [31] reported that a starvation treatment for 24 h affected the bacterial endospores amount in mealworm larvae reared on wheat bran supplemented with potato slices as a water source without influencing the other microorganisms.

Moreover, starvation also resulted effective in the reduction of *Salmonella enterica* and *Listeria monocytogenes* in mealworm larvae reared in artificially contaminated substrates [32,33]. Thus, the effect of the starvation process seems to vary in relation to the diet as well as the response of different tested bacteria.

### 3.3. Antioxidant Status

Antioxidant capacity of mealworm larvae is reported in Table 4. 

No significant differences were found among the experimental diets. However, the C diet seems to slightly improve the antioxidant capacity of the larvae with respect to the other groups (as highlighted by ABTS and FRAP values, even if no statistical significances were determined). Such a trend is justified also by the significantly higher concentration of tocopherols (mainly due to the α-isoform amount) found in the cookies-supplemented groups (C, SG-C, and B-C larvae). Nevertheless, vitamin E being a fat-soluble vitamin, it is not surprising that its concentration was higher in the mealworms with a higher fat content (ether extract values). To the best of our knowledge, this is the first evaluation of the antioxidant capacity of *T. molitor* larvae.

The antioxidant capacity of mealworms could be very interesting in order to enhance dietary antioxidant ingestion; however, as no thermal process was applied to the samples (cooking or drying), enzymes present in the larvae body may have altered the final outcomes. Few published data reported the antioxidant concentration of insects: Finke [15] summarized that the vitamin E contents ranged from 3.3–24.0 mg/kg for mealworms, 5.3–9.1 mg/kg for superworms (*Zophobas morio*), and 8.6–69.2 mg/kg for waxworms (*Galleria mellonella*). In the present study, the total vitamin E concentration of mealworms ranged from 0.51 to 5.28 mg/kg, close to the lower values reported in the literature. However, a higher concentration (>10 mg/kg) of vitamin E is reported to be common in the wild-caught insects with respect to the reared ones [34].

### 3.4. Principal Component Analysis

**Figure 2:**
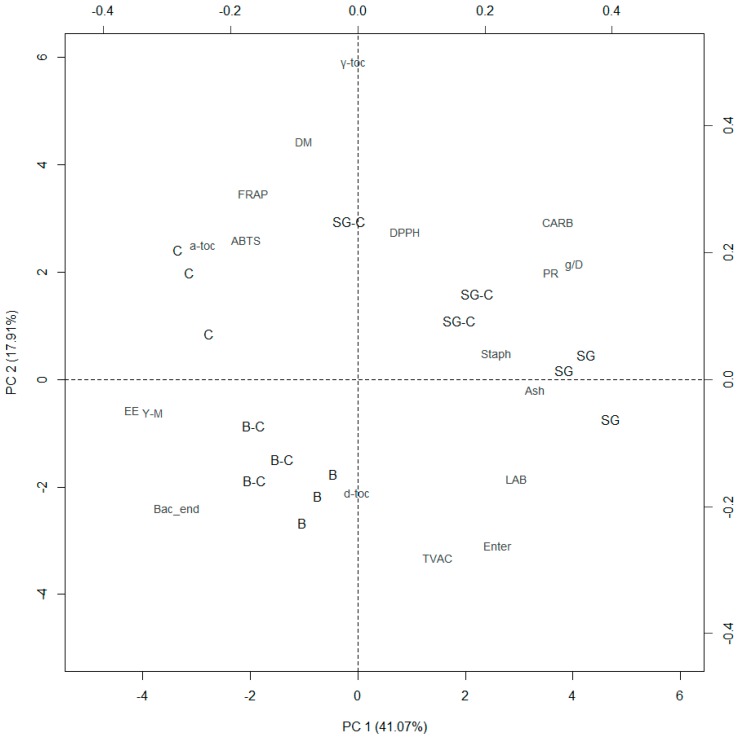
Biplot of the principal component (PC) analysis of mealworm larvae fed different diets (SG: brewery spent grains; B: bread; C: cookies; SG-C: 50% spent grains, 50% cookies; B-C: 50% bread, 50% cookies).

A principal component analysis of proximate composition, microbial loads, growth performance, antioxidant capacity, and antioxidant compounds was performed in order to detect the principal components that better describe the modifications highlighted (Figure 2).

Eigenvalues, eigenvectors, and cumulative % of the first three principal components are reported in Table 5. The first two principal components (PC1: 41.07% and PC2: 17.91%) well differentiate the samples in relation to the diet.

Positives PC1 eigenvectors collocate crude proteins on the right side of the biplot, near to SG and SG-C samples, as well as the growth performance. This relation highlights that diets rich in proteins (SG and SG-C) could promote growth and increase the protein content of the larvae. On the other hand, negative PC1 eigenvectors collocated ether extract content on the left side of the biplot, in contraposition with the protein content and the growth performances. The presence of the cookies in B-C and SG-C diets induced a shift on the left side of the plotted samples and then evidence a worsening of the growth performances and a higher deposition of fats.

Almost all the parameters that showed a statistical significance were plotted in the upper right or the lower left squares (both eigenvectors, positives or negatives) except for dry matter and tocopherol γ- and α-isoforms, which were plotted in the upper left square highlighting that another undetermined effect plays a role. As PC2 differentiates the antioxidant capacity and the tocopherol γ- and α-isoforms in the upper part of the plot and the microbial loads in the lower part, it could possible to hypothesize that a higher content of antioxidants played a role against microbial loads.

The observations reported above suggest that PC1 and PC2 were both a part of the diet effect related to the chemical composition of the substrates.

A residual percentage of variance (13.01%) was expressed also by PC3; this principal component was mostly related to (as absolute values) the antioxidant capacity and tocopherols δ- and α-isoforms.

## 4. Conclusions

In conclusion, FFPs could be employed as feed in *T. molitor* rearing with effects on the chemical composition of the larvae and growth performance. Results point out a high plasticity of mealworm larvae and the potential to tailor the final outcomes. Single FFPs or mixes of multiple FFPs could be employed in relation to the requests and benefit–cost criteria determined by the producers. In general, it seems that feeds with high protein contents determine a better performance in rearing time, at the expense of the lipid content of the larvae.

## Figures and Tables

**Table 1:** Proximate composition of brewery spent grains (SG), bread (B) and cookies (C).

Item	Unit	SG	B	C
Dry matter (DM)	%	94.81	97.09	99.99
Ether extract	%	3.29	0.31	10.44
Crude protein ^1^	%	17.98	11.15	6.55
Ash	%	3.43	1.88	0.70
Carbohydrates	%	70.10	83.75	82.29
Ether extract	% on DM	3.47	0.32	10.45
Crude protein ^1^	% on DM	18.97	11.49	6.55
Ash	% on DM	3.62	1.93	0.70
Carbohydrates	% on DM	73.94	86.26	82.30

^1^ Conversion factors of 5.83 for SG [18], of 5.70 for B, and 6.25 for C [19] (protocol numbers 950.36 and 935.39, respectively).

**Table 2:** Proximate compositions of the larvae reared on the different substrates.

Item	Unit	Rearing Substrates					RMSE	*p*-Value
		SG	B	C	SG-C	B-C		
Dry matter (DM)	%	33.33 ^c^	32.62 ^c^	35.55 ^b^	37.53 ^a^	35.33 ^b^	0.733	<0.001
Ether extract	%	6.46 ^d^	14.82 ^b^	17.77 ^a^	11.77 ^c^	17.48 ^a^	1.189	<0.001
Crude protein ^1^	%	17.36 ^a^	14.09 ^ab^	13.33 ^b^	17.65 ^a^	14.07 ^ab^	1.392	0.020
Crude protein ^2^	%	13.22 ^a^	10.73 ^ab^	10.15 ^b^	13.44 ^a^	10.72 ^ab^	1.392	0.020
Ash	%	1.10	0.98	0.95	1.07	1.01	0.097	0.371
Carbohydrates ^1^	%	8.40 ^a^	2.73 ^b^	3.50 ^b^	7.05 ^a^	2.76 ^b^	1.620	<0.001
Carbohydrates ^2^	%	12.54 ^a^	6.09 ^b^	6.72 ^b^	11.26 ^a^	6.12 ^b^	1.523	<0.001
Ether extract	% on DM	19.38 ^c^	45.43 ^a^	50.00 ^a^	31.35 ^b^	49.48 ^a^	3.389	<0.001
Crude protein ^1^	% on DM	51.34 ^a^	42.28 ^ab^	37.31 ^b^	46.40 ^ab^	40.96 ^ab^	3.898	0.022
Crude protein ^2^	% on DM	39.10 ^a^	32.20 ^ab^	28.41 ^b^	35.33 ^ab^	31.20 ^ab^	3.898	0.022
Ash	% on DM	3.36	3.05	2.69	2.88	2.81	0.268	0.084
Carbohydrates ^1^	% on DM	25.15 ^a^	8.28 ^b^	9.83 ^b^	18.76 ^a^	7.81 ^b^	4.523	<0.001
Carbohydrates ^2^	% on DM	37.58 ^a^	18.58 ^c^	18.77 ^c^	29.97 ^b^	17.31 ^c^	4.123	<0.001

SG: brewery spent grains; B: bread; C: cookies; SG-C: 50% spent grains, 50% cookies; B-C: 50% bread, 50% cookies. ^1^ Conversion factors of 6.25 for crude protein. ^2^ Conversion factors of 4.76 for crude protein. Means in the same row with no common superscripts (a–d) differ significantly (*p* < 0.05).

**Table 3:** Microbiological analyses of the larvae (un-starved and starved) reared on the different substrates.

Item	Rearing Substrates					RMSE	*p*-Value
	SG	B	C	SG-C	B-C		
No starvation							
Total viable aerobic counts	7.08	7.63	6.46	6.84	7.77	0.776	0.266
Enterobacteriaceae	6.31	6.44	6.33	5.87	6.30	0.502	0.688
Staphylococci	3.85 ^c^	5.96 ^a^	5.29 ^ab^	4.38 ^b^	5.19 ^ab^	0.579	0.005
Yeast and molds	3.30 ^b^	5.60 ^a^	6.31 ^a^	3.13 ^b^	5.34 ^ab^	0.944	0.001
Lactic acid bacteria	6.20	5.18	5.57	5.44	5.31	0.685	0.343
Bacterial endospores	0.00 ^d^	4.44 ^ab^	3.44 ^b^	2.05 ^c^	5.32 ^a^	0.240	<0.001
Starvation, 24 h							
Total viable aerobic counts	7.04	7.63	6.41	7.17	6.91	0.689	0.295
Enterobacteriaceae	6.36	6.01	4.96	5.52	5.55	0.744	0.211
Staphylococci	4.87	4.20	3.92	4.40	3.73	0.705	0.370
Yeast and molds	2.56 ^b^	4.45 ^a^	5.11 ^a^	3.54 ^ab^	4.44 ^a^	0.561	0.002
Lactic acid bacteria	6.10	5.91	4.91	5.73	5.13	0.666	0.168
Bacterial endospores	0.00 ^c^	3.62 ^a^	2.44 ^b^	2.36 ^b^	3.60 ^a^	0.243	0.038
Effect of starvation, *p*-Value							
Total viable aerobic counts	0.954	0.988	0.915	0.658	0.187		
Enterobacteriaceae	0.913	0.364	0.006	0.639	0.285		
Staphylococci	0.115	0.031	0.012	0.968	0.039		
Yeast and molds	0.506	0.014	< 0.001	0.598	0.021		
Lactic acid bacteria	0.835	0.271	0.250	0.653	0.629		
Bacterial endospores	-	0.120	0.003	0.312	0.009		

SG: brewery spent grains; B: bread; C: cookies; SG-C: 50% spent grains, 50% cookies; B-C: 50% bread, 50% cookies. Data were reported as log CFU/g. Means in the same row with no common superscripts (a–d) differ significantly (*p* < 0.05).

**Table 4:** Antioxidant capacity (ABTS, DPPH, and FRAP) and antioxidant compounds (tocopherols) of *Tenebrio molitor* larvae reared on different substrates.

Item	Rearing Substrates					RMSE	*p*-Value
	SG	B	C	SG-C	B-C		
Antioxidant Capacity							
ABTS	1.73	2.17	2.45	2.01	1.70	0.407	0.415
DPPH	0.35	0.30	0.34	0.30	0.28	0.042	0.504
FRAP	0.75	0.80	1.04	0.86	0.75	0.139	0.332
Antioxidant Compounds							
δ-tocopherol	0.12 ^c^	0.13 ^c^	0.18 ^c^	0.24 ^b^	0.33 ^a^	0.018	<0.001
γ-tocopherol	0.07 ^b^	0.02 ^b^	0.15 ^a^	0.17 ^a^	0.04 ^b^	0.017	<0.001
α-tocopherol	0.48 ^c^	0.35 ^c^	4.95 ^a^	2.86 ^b^	4.59 ^a^	0.055	<0.001
Total tocopherols	0.79 ^c^	0.51 ^c^	5.28 ^a^	3.16 ^b^	4.96 ^ab^	0.601	<0.001

SG: brewery spent grains; B: bread; C: cookies; SG-C: 50% spent grains, 50% cookies; B-C: 50% bread, 50% cookies. ABTS, DPPH and FRAP expressed as mmol of Trolox equivalent per kilogram of sample. Tocopherols and carotenes expressed as mg per kilogram of samples. Means in the same row with no common superscripts (a–c) differ significantly (*p* < 0.05).

**Table 5:** Eigenvalues and eigenvectors of the first three principal components (PC) of principal components analysis performed on proximate composition, microbial loads, growth performance, antioxidant capacity, and antioxidant compounds of mealworm larvae fed experimental diets.

Item	PC		
	PC 1	PC 2	PC 3
Eigenvalues	7.392	3.223	2.341
Eigenvectors			
Dry matter	−0.085	0.377	−0.269
Ether extract	−0.356	−0.049	−0.052
Crude protein	0.304	0.170	−0.133
Ash	0.279	−0.016	−0.204
Carbohydrates	0.314	0.250	−0.020
Weight gain (g/D)	0.340	0.182	−0.093
Total viable aerobic counts	0.127	−0.279	0.198
Enterobacteriaceae	0.220	−0.260	0.117
Staphylococci	0.219	0.042	0.156
Yeast and molds	−0.321	−0.053	0.117
Lactic acid bacteria	0.250	−0.154	0.221
Bacterial endospores	−0.284	−0.205	−0.041
ABTS	−0.176	0.220	0.451
DPPH	0.075	0.233	0.389
FRAP	−0.165	0.294	0.312
δ-tocopherol	−0.002	−0.180	−0.389
γ-tocopherol	−0.010	0.499	−0.064
α-tocopherol	−0.244	0.212	−0.329
Cumulative %	41.07	58.97	71.98

## References

[1] Van Huis A., Van Itterbeeck J., Klunder H., Mertens E., Halloran A., Muir G., Vantomme P. (2013). Edible Insects: Future Prospects for Food and Feed Security.

[2] Anankware J.P., Fening K., Obeng-Ofori D. (2015). Insects as food and feed: A review. Int. J. Agric. Res. Rev..

[3] Verbeke W. (2015). Profiling consumers who are ready to adopt insects as a meat substitute in a Western society. Food Qual. Prefer..

[4] Tan H.S.G., van Berg E.V., Stieger M. (2016). The influence of product preparation, familiarity and individual traits on the consumer acceptance of insects as food. Food Qual. Prefer..

[5] Hartmann C., Ruby M.B., Schmidt P., Siegrist M. (2018). Brave, health-conscious, and environmentally friendly: Positive impressions of insect food product consumers. Food Qual. Prefer..

[6] Mancini S., Moruzzo R., Riccioli F., Paci G. (2019). European consumers’ readiness to adopt insects as food. A review. Food Res. Int..

[7] Oonincx D.G.A.B., van Broekhoven S., van Huis A., van Loon J.J.A. (2015). Feed Conversion, Survival and Development, and Composition of Four Insect Species on Diets Composed of Food By-Products. PLoS One.

[8] Pinotti L., Giromini C., Ottoboni M., Tretola M., Marchis D. (2019). Review: Insects and former foodstuffs for upgrading food waste biomasses/streams to feed ingredients for farm animals. Animal.

[9] Wilkinson J.M. (2011). Re-defining efficiency of feed use by livestock. Animal.

[10] Van Broekhoven S., Oonincx D.G.A.B., van Huis A., van Loon J.J.A. (2015). Growth performance and feed conversion efficiency of three edible mealworm species (Coleoptera: Tenebrionidae) on diets composed of organic by-products. J. Insect Physiol..

[11] Azagoh C., Ducept F., Garcia R., Rakotozafy L., Cuvelier M.-E., Keller S., Lewandowski R., Mezdour S. (2016). Extraction and physicochemical characterization of Tenebrio molitor proteins. Food Res. Int..

[12] Osimani A., Garofalo C., Milanović V., Taccari M., Cardinali F., Aquilanti L., Pasquini M., Mozzon M., Raffaelli N., Ruschioni S. (2017). Insight into the proximate composition and microbial diversity of edible insects marketed in the European Union. Eur. Food Res. Technol..

[13] Dreassi E., Cito A., Zanfini A., Materozzi L., Botta M., Francardi V. (2017). Dietary fatty acids influence the growth and fatty acid composition of the yellow mealworm Tenebrio molitor (Coleoptera: Tenebrionidae). Lipids.

[14] Fasel N.J., Mene-Saffrane L., Ruczynski I., Komar E., Christe P. (2017). Diet induced modifications of fatty-acid composition in mealworm larvae (Tenebrio molitor). J. Food Res..

[15] Finke M.D. (2015). Complete nutrient content of four species of commercially available feeder insects fed enhanced diets during growth. Zoo Biol..

[16] Ramos-Elorduy J., González E.A., Hernández A.R., Pino J.M. (2002). Use of Tenebrio molito (Coleoptera: Tenebrionidae) to Recycle Organic Wastes and as Feed for Broiler Chickens. J. Econ. Entomol..

[17] Oonincx D.G.A.B., de Boer I.J.M. (2012). Environmental impact of the production of mealworms as a protein source for humans A Life Cycle Assessment. PLoS One.

[18] Merrill A.L., Watt B.K. (1973). Energy value of food: Basis and derivation. USDA. United States Dep. Agric..

[19] Association of Official Analytical Chemists (AOAC) (2012). Official Methods of Analysis.

[20] Janssen R.H., Vincken J.-P., van den Broek L.A.M., Fogliano V., Lakemond C.M.M. (2017). Nitrogen-to-protein conversion factors for three edible insects: Tenebrio molitor, Alphitobius diaperinus, and Hermetia illucens. J. Agric. Food Chem..

[21] Stoops J., Vandeweyer D., Crauwels S., Verreth C., Boeckx H., Van Der Borght M., Claes J., Lievens B., Van Campenhout L. (2017). Minced meat-like products from mealworm larvae (Tenebrio molitor and Alphitobius diaperinus): microbial dynamics during production and storage. Innov. Food Sci. Emerg. Technol..

[22] Mancini S., Preziuso G., Dal Bosco A., Roscini V., Szendro Z., Fratini F., Paci G., Szendrő Z., Fratini F., Paci G. (2015). Effect of turmeric powder (Curcuma longa L.) and ascorbic acid on physical characteristics and oxidative status of fresh and stored rabbit burgers. Meat Sci..

[23] Zaspel B.J., Csallany A.S. (1983). Determination of alpha-tocopherol in tissues and plasma by high-performance liquid chromatography. Anal. Biochem..

[24] R Core Team R: A Language and Environment for Statistical Computing.

[25] Herrero M., Thornton P.K. (2013). Livestock and global change: Emerging issues for sustainable food systems. Proc. Natl. Acad. Sci..

[26] Grabowski N.T., Klein G. (2017). Microbiology of processed edible insect products – Results of a preliminary survey. Int. J. Food Microbiol..

[27] Grabowski N.T., Klein G. (2017). Microbiological analysis of raw edible insects. J. Insects as Food Feed.

[28] Wynants E., Crauwels S., Verreth C., Gianotten N., Lievens B., Claes J., Van Campenhout L. (2018). Microbial dynamics during production of lesser mealworms (Alphitobius diaperinus) for human consumption at industrial scale. Food Microbiol..

[29] Vandeweyer D., Crauwels S., Lievens B., Van Campenhout L. (2017). Microbial counts of mealworm larvae (Tenebrio molitor) and crickets (Acheta domesticus and Gryllodes sigillatus) from different rearing companies and different production batches. Int. J. Food Microbiol..

[30] Wynants E., Crauwels S., Lievens B., Luca S., Claes J., Borremans A., Bruyninckx L., Van Campenhout L. (2017). Effect of post-harvest starvation and rinsing on the microbial numbers and the bacterial community composition of mealworm larvae (Tenebrio molitor). Innov. Food Sci. Emerg. Technol..

[31] Mancini S., Fratini F., Tuccinardi T., Turchi B., Nuvoloni R., Paci G. (2019). Effects of different blanching treatments on microbiological profile and quality of the mealworm (Tenebrio molitor). J. Insects as Food Feed.

[32] Wynants E., Frooninckx L., Van Miert S., Geeraerd A., Claes J., Van Campenhout L. (2019). Risks related to the presence of Salmonella sp. during rearing of mealworms (Tenebrio molitor) for food or feed: Survival in the substrate and transmission to the larvae. Food Control..

[33] Mancini S., Paci G., Ciardelli V., Turchi B., Pedonese F., Fratini F. (2019). Listeria monocytogenes contamination of Tenebrio molitor larvae rearing substrate: Preliminary evaluations. Food Microbiol..

[34] Arnold K.E., Ramsay S.L., Henderson L., Larcombe S.D. (2010). Seasonal variation in diet quality: antioxidants, invertebrates and blue tits Cyanistes caeruleus. Biol. J. Linn. Soc..

